# Exercises on Chapter First

**Published:** 1883-07

**Authors:** 


### EXERCISES ON CHAPTER FIRST.

What are the two general divisions of chemistry?

Of what does chemistry essentially treat.

Into what great divisions may science be divided?

To which of these does chemistry belong?

Give the true distinction of physical from natural science?

To which class do botany, mineralogy, and anatomy belong?

What is science?

*Frankland.

Into what three branches of study may science be conveniently divided ?

What is matter ?

Under how many aspects is matter to be considered and what are they ?

What is a mass ?

What is a molecule ?

What is an atom ?

Do atoms differ from each other and how ?

What is atomic weight ?

What is the meaning of quality of combining power ?

How does this classify atoms ?

Explain the electro-chemical relation subsisting among the chemical elements ?

Are chemical attraction and electrical attraction identical ?

Is this relation of the elements, then, relative or absolute ?

Has this principle any connection with chemical notation and nomenclature ?

Explain its relations to the chemical bodies called acids and bases ?

Give examples ?

What is meant by quantity of combining power of the atom ?

What is the standard of this power ?

If the combining power of hydrogen be 1, what numbers will express the combining powers of all other elements ?

What, then, is quantivalence ?

Is this a law of chemistry ?

Is there any deviation from this law ?

Explain and illustrate the variation of quantivalence ?

Has this variation a law ?

State it ?

By what arithmetical series may the whole of quantivalance be expressed ?

Are these series mutually convertible ?

Were atomic weight and quantivalence known to the old chemistry ?

How did the numerical equivalents of the old chemistry differ from atomic weight?

Define chemistry?

